# Childhood cardiovascular morphology and function following abnormal fetal growth

**DOI:** 10.1007/s00380-022-02064-5

**Published:** 2022-04-15

**Authors:** Rasmus F. W. Olander, Linda Litwin, Johnny K. M. Sundholm, Taisto Sarkola

**Affiliations:** 1 Children’s Hospital, Pediatric Research Center, University of Helsinki and Helsinki University Hospital, Stenbäckinkatu 9, POB 347, 00029 Helsinki, Finland; 2 Minerva Foundation Institute for Medical Research, Helsinki, Finland; 3 Department of Congenital Heart Defects and Pediatric Cardiology, FMS, Medical University of Silesia, Katowice, Poland

**Keywords:** Body composition, Cardiovascular disease; child health, Echocardiography, Fetal growth restriction

## Abstract

Studies examining the link between abnormal fetal growth and cardiac changes in childhood have presented conflicting results. We studied the effect of abnormal fetal growth on cardiac morphology and function during childhood, while controlling for body size, composition and postnatal factors. We report on the follow-up of 90 children (median age 5.81 years, IQR 5.67; 5.95) born appropriate for gestational age (AGA, *N* = 48), small for gestational age (SGA, *N* = 23), or large for gestational age (LGA, *N* = 19); SGA and LGA defined as birth weight *Z*-score < − 2 and >  + 2, respectively. We examined the heart using echocardiography, including Doppler and strain imaging, in relation to anthropometrics, body composition, blood pressure, physical activity, and diet. Although groupwise differences in body size decreased during the first year after birth, LGA remained larger at follow-up, with higher lean body mass and BMI, while SGA were smaller. Slight changes in left ventricular diastolic function were present in SGA and LGA, with SGA showing increased mitral diastolic E- and A-wave peak flow velocities, and increased septal *E*/*E*′ ratio, and LGA showing larger left atrial volume adjusted for sex and lean body mass. In univariate analyses, lean body mass at follow-up was the strongest predictor of cardiac morphology. We found no groupwise differences at follow-up for ventricular sphericity, cardiac morphology adjusted for lean body mass and sex, or blood pressure, diet, or physical activity. Cardiac morphology is predicted by lean body mass during childhood, even in the setting of abnormal fetal growth. Our results are consistent with a limited effect of fetal programming on cardiac dimensions during childhood. Minor changes in diastolic function are present in both SGA and LGA children, however, the clinical significance of these changes at this stage is likely small.

**Supplementary Information:**

The online version contains supplementary material available at 10.1007/s00380-022-02064-5.

## Introduction

The antenatal milieu affects health in adulthood [1], and low birth weight or being born small for gestational age (SGA), is linked with increased adult cardiovascular risk [2]. Being born large for gestational age (LGA) associates with obesity [3] and a U-shaped association between cardiovascular risk and birth weight has been observed [4].

Conflicting findings on the link between fetal growth and the cardiovascular system in childhood have been presented. SGA and fetal growth restriction (FGR) have been linked to cardiac remodeling in childhood [5]. This remodeling, observed as increased ventricular sphericity, has been reported in 5-year-olds [6] and preadolescents [7], along with altered systolic [7] and diastolic function [6]. Birth weight associates with ventricular mass in early childhood [8] and adolescence [9], with higher birth weight associated with changes in diastolic function [10]. Recent studies have, however, demonstrated that SGA children show size-appropriate cardiovascular dimensions in early childhood [11, 12], suggesting a limited impact of altered fetal growth. We have previously demonstrated cardiac morphology of SGA and LGA neonates to be appropriate for body size, with no evidence of altered geometry [13].

Body surface area (BSA) is commonly used to adjust childhood cardiac dimensions for body size. The association between cardiac structures and BSA is often not linear [14], and indexing with BSA raised to different exponents have been suggested as alternatives [15]. Lean body mass (LBM) has been proposed as the main predictor of cardiovascular morphology [16] and left ventricular mass [17]. We have previously discussed that adjusting for BSA could lead to over-adjusting cardiac dimensions in the presence of adiposity [13], and shown LBM to be the strongest predictor of left ventricular mass during childhood [18]. Taking current body size into account is especially important when examining children born SGA and LGA, as both SGA [12] and LGA [19] are associated with changes in body composition.

We aimed to assess the effect of fetal growth on cardiac morphology, geometry, and function in children born either LGA or SGA with FGR, and to explore associations between cardiovascular morphology, body size and composition, while controlling for postnatal factors, diet and physical activity. We hypothesized cardiovascular morphology to associate with current body size, with LBM being the main predictor.

## Materials and methods

### Study design, sample and setting

In this study, we report on the follow-up of 90 patients [appropriate for gestational age (AGA) *N* = 48, SGA *N* = 23, LGA *N* = 19], examined between October 2017 and June 2019. Initially, we examined a cohort of 174 newborns born at gestational weeks 31–42 between November 2011 and January 2014 at the Women’s Hospital, Helsinki, Finland. We recruited the children into three groups at birth: SGA, LGA and appropriate for gestational age (AGA). The groups were defined according to weight *Z*-score for gestational age at birth according to Finnish growth charts [20] in use at that time, with SGA defined as < − 2 and LGA >  + 2. The birthweight of the SGA group was below the 3rd percentile, corresponding to the criteria for FGR, as set by the International Society of Ultrasound in Obstetrics and Gynecology practice guidelines [21]. Inclusion and exclusion criteria and gestational data for the initial recruitment have been reported earlier [13, 22]. None of the patients who agreed to the follow-up were excluded.

New Finnish fetal and childhood growth references were published during the follow-up time, and we generated new *Z*-scores for birth weight [23, 24]. These new *Z*-scores corresponded well to group assignment at birth, with only minor discrepancies, and no reassignment was done between the groups as to maintain compatibility with the newborn stage. Written informed consent was given by the children’s guardians at both baseline and follow-up enrollment. The Helsinki University Hospital Ethics Committee for gynecology and obstetrics, pediatrics and psychiatry approved the research protocol (138/13/03/03/2011 and HUS/2274/2016).

### Anthropometrics, body composition, blood pressure, diet and physical activity

At the follow-up visit, we measured standard anthropometrics (Seca285, Seca GmBH & Co. KG, Hamburg, Germany), with thoracic circumference measured using a tape measure at the level of the xiphoid process. BSA was calculated using the Haycock formula [25]. We assessed body composition using bioelectrical impedance analysis (InBody 7250, Inbody Bldg., Seoul, South Korea). Growth data were obtained from primary health care centers. We generated *Z*-scores for height and BMI in relation to age and for weight in relation to height [24]. Prior to the visit, a food frequency questionnaire was filled in by the children’s parents and after the visit physical activity was monitored for one week with an accelerometer (ActiGraph wGT3X-BT, ActiGraph LLC, Pensacola, FL, USA), as outlined in Supplementary methods (Online Resource 1). Body composition data were obtained for 89 children, dietary patterns in 85, and physical activity monitored for 83.

Blood pressure was measured, following a 1-h rest period, in the upright sitting position from the right arm by a trained technician using appropriately sized cuffs (Carescape v100, GE Healthcare, Chicago, USA). Means from three consecutive measurements were used in analysis. Parallel measurement coefficients of variance (CV) were 4% for systolic and diastolic blood pressure, and 6% for heart rate.

### Echocardiography

One investigator examined the children with transthoracic echocardiography using a 7-MHz 7S transducer (Vivid 7, General Electric Medical Systems, Horten, Norway). Another investigator performed the measurements offline (EchoPac version 204, General Electric) from standard views, according to American Society of Echocardiography recommendations [26, 27]. Arterial diameters were measured in systole, except for the abdominal aorta which was examined in diastole. We measured the inferior vena cava at its maximum in expiration and minimum in inspiration and calculated the percentage change.

Left ventricular mass was attained from parasternal short axis B-mode measurements using Devereux’s formula [28]. We determined left ventricular systolic and diastolic volumes using the Simpson biplane technique. Left atrial volume was assessed with the biplane area–length method at ventricular end-systole. Sphericity indices were calculated by dividing diastolic 4-chamber ventricular lengths with diastolic short axis diameters (left ventricular base or right ventricular base and mid-cavity diameters from 4-chamber view and left ventricular diastolic dimension from the parasternal short axis view at the mid-papillary level).

We assessed the systolic function of the ventricles by calculating the ejection fraction and fractional area change of the left and right ventricles respectively, by measuring the mitral and tricuspid annular plane systolic excursion (MAPSE and TAPSE) with M-mode, and through standard myocardial tissue Doppler and speckle tracking strain measurements. We examined diastolic function through standard pulsed wave and tissue Doppler and strain measurements, and by examining left atrial volume as noted above. Echocardiograms were obtained for 88 of the children. Intra- and inter-observer variability (CV) was assessed for morphologic measurements, ranging from 4 to 8% and 5 to 10%, respectively.

### Statistical analysis

We present results as count, mean ± SD, median Q1; Q3 or adjusted mean (SE). Normality was assessed from histograms and using the Shapiro–Wilk test. We used ANOVA, Kruskal–Wallis, and Fisher–Freeman–Hamilton exact tests to compare unadjusted values between groups, with corresponding post hoc tests (Dunnet or Games–Howell, Mann–Whitney *U*, *Z*-test for proportions). Adjusted means were examined using ANCOVA, with Fisher’s LSD used post hoc. A two-sided *P* value < 0.05 was determined statistically significant. Significance levels of Mann–Whitney *U*, *Z*-test for proportions and Fisher’s LSD were Bonferroni-corrected for two pairwise comparisons, as group differences between AGA and SGA or LGA were examined.

We first compared background, cardiac morphology, and function between the three groups. We then assessed the effect of sex, birth weight *Z*-score, current body size and age on cardiac morphology using Student’s *t* test and Pearson’s correlation analysis for the normally distributed variables. For the non-normally distributed left ventricular septal and posterior wall dimensions and the right ventricle anterior wall dimensions, we used the Mann–Whitney *U* test and Spearman’s correlation analysis. We chose to examine height, weight, thoracic circumference, LBM and BSA as predictors describing body size, along with BSA and height raised to exponents, as suggested by Lopez et al. [15], Bhatla et al. [29] and Chinali et al. [30] for arterial and left atrial and ventricular dimensions, mass, and volumes. For right ventricular dimensions, we used BSA^0.50^. We examined BMI and body fat percentage as predictors describing adiposity. BMI was chosen instead of BMI *Z*-score due to the homogenous age of the sample. We further assessed the role of maternal pre-gestational diabetes and systolic blood pressure as potential confounders for left ventricular mass.

We identified sex as a potential confounder affecting both cardiovascular dimensions and body size, and current body size and adiposity as mediators. We explored ANCOVA models for cardiac morphology to adjust groupwise cardiac morphology for the effect of sex, current body size and adiposity, and to compare LBM, BSA or BSA raised to an exponent as predictors. We used LBM, BSA or BSA raised to an exponent as markers of current body size and explored adiposity by adding BMI as a predictor to the models adjusted for sex and LBM. Due to the non-normal distribution of residuals, models for left ventricular septal and posterior wall dimension and right ventricular anterior wall dimensions are not presented.

Finally, multiple linear regression models with sex, birth weight *Z*-score and LBM as predictors were created, to explore birth size as a continuous variable. We examined the linear regression models for multicollinearity and chose a variance inflation factor cut-off value of 2.5. We used SPSS 27 (IBM, New York, USA) for data analysis, and created graphs using GraphPad Prism 9 (GraphPad Software, San Diego, California, USA).

## Results

### Background and anthropometrics

Significant differences were observed between groups in anthropometry, with the SGA children consistently reporting smaller body size, including height, weight, and LBM, but similar adiposity to AGA (Table 1). The LGA children were overall larger, with higher weight, LBM and BMI, suggesting higher adiposity. Body fat percentage did not vary significantly between groups. The proportion of pre-gestational diabetes was higher in the LGA group, and pre-eclampsia was more common in the SGA group. We observed no differences in dietary patterns between the groups, and physical activity between the groups was similar (Supplementary Table 1, Online Resource 1). We found no differences in light, moderate or vigorous physical activity between the groups (Supplementary Table 1, Online Resource 1), while the LGA group reported a slightly, yet statistically significant, higher time spent in bed.

**Table 1:** Background and anthropometric data for the AGA, SGA, LGA groups at follow-up

	AGA	(*N* = 48)	SGA	(*N* = 23)	LGA	(*N* = 19)	*p*
Maternal pre-pregnancy diabetes	5		0		14***		< 0.001
Maternal pre-eclampsia	3		8**		1		0.003
Child sex (male/female)	27/21		9/14		9/10		0.376
Gestational age (weeks)	34.9	34.1; 39.3	37.6	34.6; 38.1	35.7	34.6; 37.6	0.602
Birth weight (g)	2550	2195; 3474	2005***	1735; 2390	4060***	3740; 4480	< 0.001
Birth weight (*Z*-score)	0.06	− 0.69; 0.85	− 2.67***	− 2.86; − 1.97	3.87***	3.10; 4.13	< 0.001
Weight at 1 y (*Z*-score)	− 0.03	± 1.01	− 0.98**	± 1.30	0.39	± 0.76	< 0.001
Age at follow-up (years)	5.8	5.7; 5.9	5.8	5.7; 6.1	5.8	5.7; 5.9	0.776
Height (cm)	116.8	± 4.5	111.8***	± 4.5	117.5	± 3.6	< 0.001
Height (*Z*-score)	− 0.03	± 0.90	− 1.15***	± 1.06	0.18	± 0.65	< 0.001
Weight (kg)	20.1	18.0; 22.3	17.9**	15.2; 20.7	22.6**	21.2; 23.9	< 0.001
Weight (*Z*-score)	− 0.83	± 1.28	− 1.48	± 2.02	0.38***	± 1.03	< 0.001
BMI (kg/m^2^)	14.9	14.0; 15.7	13.9	13.1; 15.9	16.1**	15.3; 16.9	< 0.001
BMI (*Z*-score)	− 0.80	± 1.29	− 1.38	± 1.92	0.38***	± 1.04	< 0.001
Body surface area (m^2^)	0.81	± 0.07	0.75**	± 0.08	0.86*	± 0.08	< 0.001
Thoracic circumference (cm)	56.4	54.0; 57.8	53.5**	50.0; 56.1	57.7	55.5; 61.0	< 0.001
Lean body mass (kg)	17.7	± 1.9	15.8***	± 1.8	18.9*	± 1.6	< 0.001
Body fat (%)	13	9; 16	8	4; 15	15	12; 18	0.046
Systolic BP (mmHg)	101	± 7	102	± 10	100	± 6	0.790
Systolic BP (*Z*-score)	0.65	± 0.67	0.85	± 0.88	0.53	± 0.66	0.334
Diastolic BP (mmHg)	60	56; 65	58	56; 65	58	55; 62	0.780
Diastolic BP (*Z*-score)	0.31	0.00; 0.95	0.36	0.15; 0.99	0.28	− 0.08; 0.61	0.538
Heart rate (bpm)	84	± 11	83	± 12	81	± 8	0.514

Data are given as mean ± SD, median Q1; Q3 or count. *P* correspond to ANOVA, Kruskal–Wallis or Fisher–Freeman–Halton exact test, as appropriate. Significant differences in post hoc tests (Dunnet, Games–Howell, Mann–Whitney *U*, or *Z* test for proportions) between SGA or LGA and AGA in are indicated with *, **, *** corresponding to two-sided significance levels of < 0.05, < 0.01, and < 0.001. respectively. The significance levels of Mann–Whitney *U* test and *Z* test for proportions are Bonferroni-corrected for two group comparisons

*AGA* appropriate for gestational age; *BP* blood pressure; *LGA* large for gestational age; *SGA* small for gestational age

When examining children participating and not participating in the follow-up, we found no differences for sex, gestational parameters, birth weight Z-score or distribution of children between the SGA/LGA/AGA groups (results not shown).

### Cardiovascular morphology and function unadjusted for sex and body size

When comparing unadjusted cardiovascular morphology groupwise, the SGA group showed significantly smaller diameters at the aortic valve and isthmus, smaller diameters at the bases of both ventricles, and smaller right ventricular and atrial areas (Supplementary Table 2, Online Resource 1). LGA showed an increased left atrial volume. No differences in sphericity of either ventricle were observed between groups (Table 2).

**Table 2:** Cardiac geometry and function for the AGA, SGA and LGA groups

	AGA	(*N* = 48)	SGA	(*N* = 23)	LGA	(*N* = 19)	*p*
*Geometry of ventricles*							
Left ventricular basal sphericity index (no unit)	2.1	± 0.2	2.2	± 0.2	2.1	± 0.2	0.185
Left ventricular mid-papillary sphericity index (no unit)	1.5	1.4; 1.5	1.5	1.3; 1.6	1.5	1.4; 1.6	0.432
Right ventricular base sphericity index (no unit)	1.9	± 0.2	2.0	± 0.2	1.9	± 0.2	0.202
Right ventricular mid-cavity sphericity index (no unit)	2.1	± 0.2	2.1	± 0.2	2.1	± 0.2	0.634
*Diastolic function*							
Mitral E-wave peak velocity (cm/s)	87	80; 99	99*	90; 110	87	81; 98	0.013
Mitral A-wave peak velocity (cm/s)	40	32; 50	49**	42; 55	41	34; 53	0.014
Mitral E/A ratio (no unit)	2.3	1.8; 2.7	1.9	1.8; 2.2	2.1	1.8; 2.7	0.461
Septal *E*′-wave peak velocity (cm/s)	13	± 1	13	± 1	13	± 1	0.683
Septal *E*/*E*′ ratio (no unit)	6.6	6.0; 8.1	7.5*	7.0; 8.3	6.7	6.1; 7.0	0.007
*Systolic function*							
Left ventricular ejection fraction (%)	58	± 3	57	± 4	58	± 3	0.408
Mitral annular plane systolic excursion (cm)	1.4	± 0.2	1.3*	± 0.2	1.5	± 0.2	0.003
Mitral annular plane systolic excursion, indexed^a^ (no unit)	0.26	± 0.03	0.24	± 0.02	0.26	± 0.04	0.032
Right ventricular fractional area change (%)	44	40; 49	41	39; 44	42	40; 46	0.129
Tricuspid annular plane systolic excursion (cm)	2.0	± 0.2	1.8*	± 0.2	2.0	± 0.19	0.028
Tricuspid annular plane systolic excursion, indexed^a^ (no unit)	0.40	± 0.05	0.38	± 0.05	0.39	± 0.06	0.333

Data are given as mean ± SD or median Q1; Q3. *P* correspond to ANOVA or Kruskal–Wallis test, as appropriate. Significant differences in post hoc tests (Dunnet or Mann–Whitney *U*) between SGA or LGA and AGA are indicated with * and **, corresponding to two-sided significance levels of < 0.05 and < 0.01, respectively. The significance level of Mann–Whitney *U* test is Bonferroni-corrected for two group comparisons. *AGA* appropriate for gestational age; *LGA* large for gestational age: *SGA* small for gestational age

^a^Divided by corresponding ventricle length

The SGA group displayed increased mitral E- and A-wave peak velocities (Table 2), reflected as significantly increased septal *E*/*E*′ ratio. The SGA group also showed a slightly decreased MAPSE and TAPSE. However, these differences disappeared once adjusted for ventricular length. No other differences in function, as measured with B mode (Table 2), Doppler (Supplementary Table 3, Online Resource 1), or using strain imaging (Supplementary Table 4, Online Resource 1) were observed.

### Cardiovascular morphology adjusted for sex and current body size

**Fig. 1:**
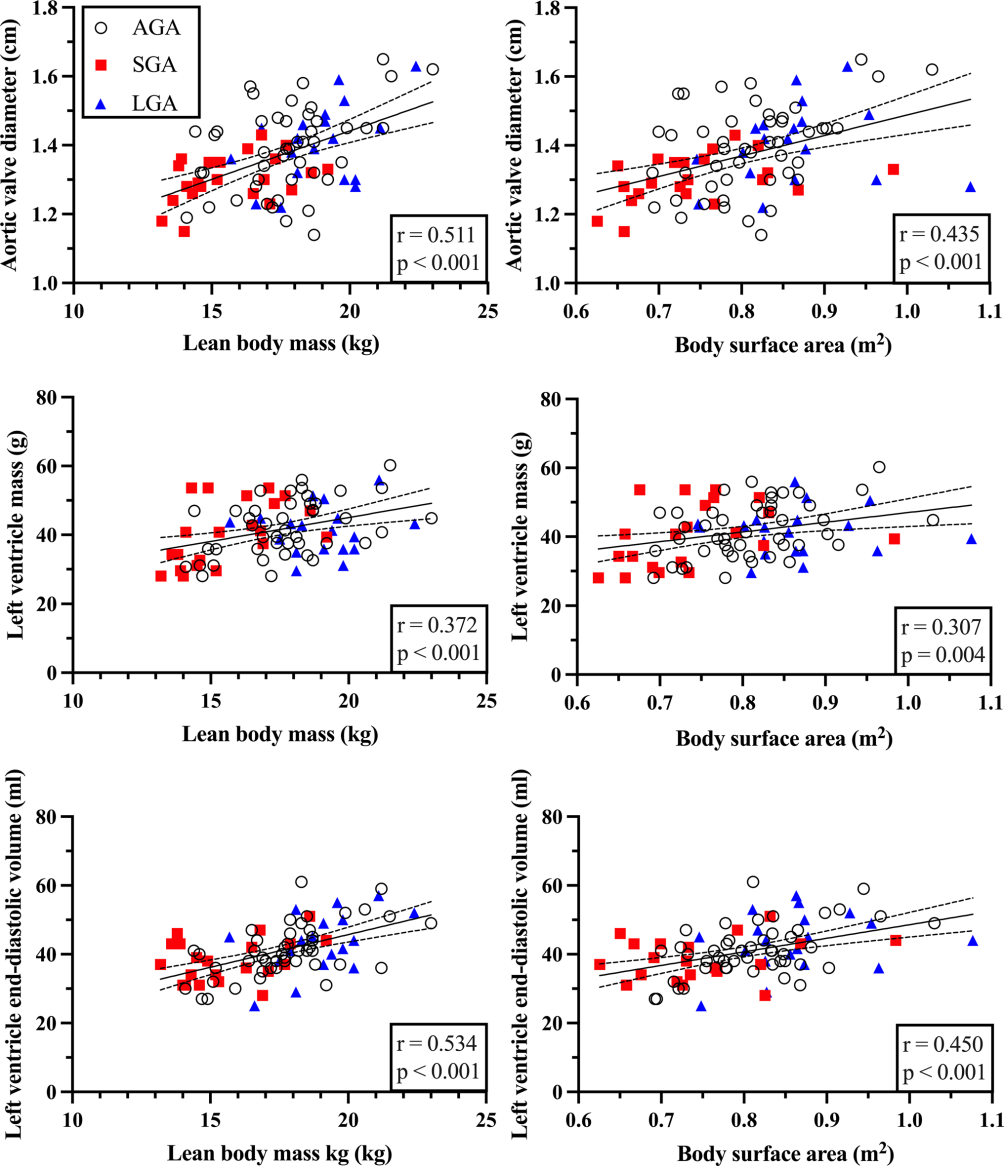
Aortic valve diameter, left ventricular mass and volume plotted against lean body mass and body surface area. AGA indicates appropriate for gestational age, SGA small for gestational age, LGA large for gestational age. Data are presented with a linear regression line with 95% confidence interval bands, and with Pearson’s correlation coefficient, r

In univariate analyses (Supplementary Table 5, Online Resource 1), we observed significant associations between cardiovascular morphology, sex, birth weight, current body size and adiposity. Boys showed larger aortic valve and sinus diameter, left ventricular diastolic diameter, mass and volume, along with right ventricular mid-cavity diameter and diastolic area. LBM was the strongest and most consistent predictor of cardiovascular morphology (Fig. 1) (*r* = 0.265–0.534, *p* < 0.05). No association was observed between cardiovascular morphology and age, or systolic blood pressure and maternal pre-gestational diabetes and left ventricular mass (not shown).

In the ANCOVA models, we examined morphology groupwise, adjusted for sex and LBM, BSA or BSA raised to an exponent. We also added BMI as a predictor of adiposity to the models with LBM (data not shown). Models adjusting groupwise differences for only sex and LBM performed the best, with higher adjusted R^2^ than models with sex and BSA or BSA raised to an exponent. The only exception was the diastolic abdominal aorta diameter, for which both models with BSA and BSA^0.50^ slightly outperformed LBM (adjusted *R*^2^ 0.142 and 0.140 respectively, vs 0.112). Adding BMI to the LBM model did not result in overall improvements in model fit, except for abdominal aorta diameter and right ventricular basal diameter (adjusted *R*^2^ 0.158 vs 0.142 and 0.165 vs 0.149), with BMI only remaining independently associated with abdominal aorta diameter. As no independent association was observed for the other dimensions, and BMI did not alter the groupwise differences of the abdominal aorta, only models without BMI are reported (Table 3). Adjusting for sex and LBM removed most significant groupwise differences, with only left atrial volume remaining increased for the LGA group (Table 3).

**Table 3:** Cardiovascular morphology for the AGA, SGA and LGA groups with adjusted means for sex and lean body mass

	AGA	(*N* = 48)	SGA	(*N* = 23)	LGA	(*N* = 19)	*p*
*Aorta and pulmonary artery*							
Abdominal aorta diameter (cm)	0.80	(0.01)	0.79	(0.02)	0.83	(0.02)	0.218
Aortic proximal arch diameter (cm)	1.53	(0.03)	1.51	(0.05)	1.51	(0.05)	0.900
Aortic distal arch diameter (cm)	1.19	(0.02)	1.22	(0.03)	1.17	(0.03)	0.544
Aortic isthmus diameter (cm)	1.11	(0.02)	1.07	(0.04)	1.12	(0.04)	0.661
Aortic valve diameter (cm)	1.38	(0.02)	1.35	(0.02)	1.34	(0.03)	0.574
Aortic root diameter (cm)	1.86	(0.03)	1.87	(0.04)	1.84	(0.05)	0.877
Aortic sinotubular junction diameter (cm)	1.61	(0.02)	1.67	(0.040)	1.62	(0.04)	0.360
Pulmonary valve diameter (cm)	1.90	(0.03)	1.82	(0.05)	1.86	(0.05)	0.437
*Left ventricle and atrium*							
Left ventricular end-diastolic diameter (cm)	3.7	(0.0)	3.8	(0.1)	3.6	(0.1)	0.049
Left ventricular mass (g)	41	(1)	44	(2)	39	(2)	0.254
Left ventricular length (cm)	5.5	(0.1)	5.6	(0.1)	5.5	(0.1)	0.553
Left ventricular base (cm)	2.6	(0.0)	2.5	(0.0)	2.6	(0.0)	0.783
Left ventricular end-diastolic volume (ml)	40	(1)	42	(2)	41	(2)	0.715
Left atrial volume (ml)	18	(1)	19	(1)	20*	(1)	0.036
*Right ventricle and atrium*							
Right ventricular length (cm)	4.9	(0.1)	4.9	(0.1)	5.0	(0.1)	0.682
Right ventricular base (cm)	2.6	(0.0)	2.5	(0.0)	2.7	(0.1)	0.304
Right ventricular mid-cavity diameter (cm)	2.4	(0.0)	2.4	(0.1)	2.3	(0.1)	0.694
Right ventricular diastolic area (cm^2^)	10.3	(0.2)	10.2	(0.3)	10.2	(0.3)	0.903
Right atrial area (cm^2^)	7.5	(0.2)	7.2	(0.2)	7.6	(0.2)	0.442

Data are given as adjusted mean (SE). *P* corresponds to group as a predictor in ANCOVA. Significant differences in post hoc tests (Fisher's LSD) between AGA and SGA or LGA are indicated with * corresponding to a two-sided significance level of < 0.05. The significance level of Fisher's LSD is corrected for two group comparisons

*AGA* appropriate for gestational age; *LGA* large for gestational age; *SGA* small for gestational age

Notably, in ANCOVA models with LBM, sex remained a significant independent predictor only for left ventricular diastolic diameter and right ventricular mid-cavity diameter and diastolic area, while in the models containing BSA or BSA raised to an exponent, sex remained a significant independent predictor for aortic valve and sinus, left ventricular mass and end-diastolic volume, in addition to left ventricular diastolic diameter and right ventricular mid-cavity diameter and diastolic area. When examining differences for BSA and LBM for sex, no difference was found for BSA, while boys had a significantly larger LBM (mean difference 1.1 kg, *p* = 0.018). No significant interaction was observed in any of the models between group and current body size.

Based on these findings, multiple linear regression models with birth weight, sex and LBM as predictors were constructed (Supplementary Table 6, Online Resource 1). LBM remained an independent predictor for all measures, except for abdominal aorta dimension. Sex remained an independent predictor of right ventricular mid-cavity diameter and diastolic area and left ventricular diastolic diameter, similar to ANCOVA models.

## Discussion

In this study, we examined the role of prenatal growth, current body size and composition, and the influence of a comprehensive set of other potential factors on the morphology and function of the heart during childhood. Despite reporting subtle changes in left ventricular diastolic function, we found no evidence of changes in cardiac morphology or geometry attributable to restricted or excessive fetal growth and body size at birth. Overall, our findings suggest that cardiac morphology during childhood is largely determined by current LBM and sex in the setting of abnormal fetal growth.

The effect of altered fetal growth on cardiac geometry during childhood seems to be small. In contrast to our findings, increased sphericity of the ventricles of SGA and FGR fetuses [31], neonates [32], and children [6, 7], have been reported previously. We and other studies have found no direct signs of increased globularity of the ventricles, in neonates [11, 13] or during childhood [12], suggesting that any impact of SGA or FGR on the sphericity of the ventricle is limited, and not present in all cases of SGA. This is supported by findings suggesting only subtle cardiac changes in SGA adults [33].

While we found significantly increased diastolic mitral E- and A-wave peak inflow velocities, and an increased septal *E*/*E*′ ratio in the SGA group, possibly indicating differences in ventricular filling, these differences were minor. Previous studies report both increased [6] and similar [12] *E*/*E*′ ratios in SGA children during early childhood, while in the neonate stage, we found the *E*/*E*′ ratio to be decreased in SGA and increased in LGA [13]. The increased left atrial volume observed in the LGA group suggests that diastolic function could potentially be affected by high birth weight. The slight changes could be due to differences in current anthropometrics, and we have previously found left atrial volume to significantly associate with body fat percentage in children in the same age group [34]. Thus, abnormal fetal growth seems to affect diastolic function in early childhood. The differences are, however, minor, and their clinical significance likely small at this stage.

We found that even in the setting of abnormal fetal growth, cardiovascular dimensions in early childhood are largely explained by current body size, LBM and sex. This is supported by our previous findings in children examining left ventricular mass in the presence of maternal obesity and diabetes, which found LBM to be the strongest predictor of left ventricular mass [18]. Interestingly, we found LBM, in comparison to BSA, to attenuate the independent association between sex and cardiac dimensions. This suggest that relations between cardiac dimensions and sex are largely mediated by sex differences in LBM. This implies, that once adjusted for LBM, the influence of sex on cardiovascular dimensions is limited in early childhood. Earlier studies have also found LBM to remove sex differences in left ventricular mass [35]. The relationship between LBM and cardiac dimensions is not necessarily similar for all dimensions, as suggested by BSA outperforming LBM for the abdominal aorta dimension. This could be due to adiposity influencing these dimensions to some extent, as suggested by the independent association between BMI and the abdominal aorta dimension in the ANCOVA models. The impact of adiposity on arterial dimensions is, however, likely small, as we have previously demonstrated that arterial dimensions overall, and lumen diameters in particular, most strongly associate with LBM in preschool children [36]. Further studies are, needed, to determine to what extent adjusting for LBM removes the need to account for sex in cardiac dimensions.

Our study is mainly limited by the lack of differentiation regarding time-of-onset of FGR. The severity of FGR has previously been found to influence cardiac morphology in fetuses, with early-onset FGR being associated with hypertrophy and late-onset with elongation or increased sphericity of the ventricles in fetuses [37]. Furthermore, the small sample size limits the conclusions we can draw on LBM as a predictor of cardiac dimensions in the general pediatric population. Bioimpedance is also known to underestimate fat mass in childhood [38], limiting comparison to LBM derived with other methods. The strengths of our study are the well-documented anthropometrics, the comprehensiveness of the assessment overall, including blood pressure and physical activity potentially influencing the heart, and the longitudinal cohort sample. Finally, the firm inclusion criteria for SGA and LGA, i.e., the most extreme 2.3 percentiles of body size at birth, together with the documented normalization of body size during the first postnatal year of life, is consistent with our SGA cohort fulfilling the criteria for FGR in its entirety [21], further strengthening the generalizability of our results to populations with significant abnormal fetal growth.

## Conclusion

In this study, we show that cardiac morphology is mainly predicted by current body size and LBM in children with restricted and excessive fetal growth. While subtle changes in diastolic function were observed both in the SGA and LGA children, their clinical significance at this stage is likely small. We found cardiac morphology to overall be appropriate for current body size. This suggests that effect of fetal growth on cardiac morphology in childhood is limited.

## Supplementary Information

Below is the link to the electronic supplementary material.

Supplementary file1 (PDF 432 kb)
