# Supplementary material for: Childhood cardiovascular morphology and function following abnormal fetal growth
Source: Heart Vessels. 2022 Apr 15;37(9):1618–27. doi: 10.1007/s00380-022-02064-5 (PMC9349157; doi:10.1007/s00380-022-02064-5)
Supplement: Supplementary file 1 — Supplementary file1 (PDF 432 kb) [file 380_2022_2064_MOESM1_ESM.pdf]

SUPPLEMENTARY MATERIAL

Re: Childhood cardiovascular morphology and function following abnormal fetal growth

Journal: Heart and Vessels

Rasmus F.W. Olander[a, b], MD

Linda Litwin[b, c], MD

Johnny K.M. Sundholm[a, b], MD, PhD

Taisto Sarkola[a, b], MD, PhD

[a]Children's Hospital, Pediatric Research Center, University of Helsinki and Helsinki University Hospital, Helsinki, Finland

[b]Minerva Foundation Institute for Medical Research, Helsinki, Finland

[c]Department of Congenital Heart Defects and Pediatric Cardiology, FMS, Medical University of Silesia, Katowice, Poland

Corresponding author: Rasmus Olander, Children's Hospital, Pediatric Research Center, University of Helsinki and Helsinki University Hospital, e-mail: rasmus.olander@helsinki.fi

SUPPLEMENTARY METHODS

Dietary patterns

Dietary patterns were examined using a food frequency questionnaire, in which the frequency of consumption of certain food items at home was recorded for one week. A modified version of the Finnish Children Healthy Eating Index (FCHEI) was used to examine these dietary patterns [1]. To create this modified FCHEI-score, we used the frequency of use of food items belonging to five major subcategories (vegetables, fruits and berries, skimmed milk, foods containing high amounts of sugar, fish and fish dishes and the use of vegetable oils or margarine). Subcategory scores were created by first assigning 0 score if subcategory frequency was 0, and then dividing those who have used a product into quantiles. For vegetables, fruits, and berries as well as foods containing high amounts of sugar, no subject scored 0, and data was divided into deciles. For skimmed milk and fish and fish dishes, subjects were divided into fewer groups due to granularity of data. For vegetable oils and margarine, the frequency of use was not recorded, and subjects were assigned a score of either 0 or 5 based on using vegetable oils or margarine, or not using either. The scores of these five subcategories were then added up to generate the complete modified FCHEI-score, and groupwise differences were examined for both the score as a whole and for the distribution of subjects within each score quartile. An experienced nutritionist was consulted when modifying the FCHEI.

Physical activity

Physical activity was investigated using the activity monitor ActiGraph wGT3X-BT, (ActiGraph LLC, Pensacola, FL, USA), and physical activity was measured for 7 consecutive days, yielding 6 days of full data. Data was recorded in 10 second epochs and analyzed using Evenson's cut-off points [2], and time spent sleeping was generated algorithmically [3]. Mean times per day, weighted for weekday and weekend days, are reported. Minimum recorded time per day, for a day to be considered valid, was set at 480 min, with 180 min of sleep required. For a measurement to be considered valid at least 2 full weekdays and 1 weekend day had to be recorded.

References

1. Kyttälä P, Erkkola M, Lehtinen-Jacks S, Ovaskainen ML, Uusitalo L, Veijola R, Simell O, Knip M, Virtanen SM (2014) Finnish Children Healthy Eating Index (FCHEI) and its associations with family and child characteristics in pre-school children. Public Health Nutr 17:2519-2527

2. Evenson KR, Catellier DJ, Gill K, Ondrak KS, McMurray RG (2008) Calibration of two objective measures of physical activity for children. J Sports Sci 26:1557-1565

3. Sadeh AV, Sharkey KM, Carskadon MA (1994) Fundamental Research Activity-Based Sleep-Wake Identification: An Empirical Test of Methodological Issues. Sleep 17:201

SUPPPLEMENTARY TABLES

Supplementary Table 1. Dietary patterns for the AGA, SGA and LGA groups according to the Finnish Healthy Eating Index, with score ranges specified for index, its quartiles, and its subcategories, along with physical activity, as measured with Actigraph, compared between groups.

	AGA	(N = 48)	SGA	(N = 23)	LGA	(N = 19)	p
Finnish Children Healthy Eating Index Score (3-26)	16	± 6	15	± 4	14	± 5	0.705
Finnish Children Healthy Eating Index Quartiles							
Quartile 1 (3-10)	12	25%	5	25%	5	29%	0.422
Quartile 2 (11-16)	12	25%	7	35%	4	24%	
Quartile 3 (17-21)	11	23%	7	35%	6	35%	
Quartile 4 (21-26)	13	27%	1	5%	2	12%	
Finnish Children Healthy Eating Indexing Subcategories							
Vegetables, fruits and berries (1-10)	5	3; 9	5	3; 8	5	2; 7	0.732
Skimmed milk (0-5)	1	0; 2	1	0; 2	0	0; 0	0.054
Foods containing high amounts of sugar (1-10)	5	3; 8	6	4; 8	8	3; 10	0.668
Fish and fish dishes (0-4)	1	1; 2	2	1; 3	1	1; 2	0.375
Vegetable oils and margarine (fat ≥ 55 %) (0 or 5)							
Vegetable oils or margarine, fat content ≥ 55% (5)	29	60%	15	75 %	12	71 %	0.500
Does not use vegetable oils or margarine, or uses low-fat versions (0)	19	40%	5	25 %	5	29 %	
Physical activity							
Wearing time (awake) (min)	784	± 41	760	± 33	757	± 56	0.032
Sedentary time (min)	428	396; 456	415	378; 454	421	376; 449	0.511
Light physical activity (min)	288	± 29	278	± 31	275	± 37	0.286
Moderate physical activity (min)	51	± 15	49	± 12	49	± 15	0.810
Vigorous physical activity (min)	21	15; 27	16	14; 26	19	15; 31	0.580
Moderate and vigorous physical activity (min)	72	± 23	69	± 19	71	± 25	0.834
Number of steps (n)	9306	8390; 11403	8878	8213; 10748	8715	7998; 10015	0.447
Time spent in bed (h)	10.1	± 0.5	10.4	± 0.4	**10.5***	± 0.7	0.040
Sleeping time (h)	9.4	9.07; 9.80	9.5	9.06; 9.90	9.7	9.2; 10.4	0.300
Moderate and vigorous physical activity of wearing time (%)	9.2	± 3.0	9.0	± 2.6	9.3	± 2.9	0.964

Data are given as mean ± SD, median Q1; Q3, or N and % within group. P correspond to ANOVA, Kruskal-Wallis, or Fisher-Freeman-Halton exact test, as appropriate. Significant differences in post-hoc tests (Dunnet) are bolded and indicated with *, corresponding to a significance level of <0.05. *AGA* appropriate for gestational age; *LGA* large for gestational age; *SGA* small for gestational age.

Supplementary Table 2. Cardiovascular morphology for the AGA, SGA and LGA groups, unadjusted.

	AGA	(N = 48)	SGA	(N = 23)	LGA	(N = 19)	p
Inferior vena cava, aorta, and pulmonary artery							
Inferior vena cava minimum diameter (cm)	0.28	0.22;0.36	0.29	0.20; 0.37	0.25	0.22; 0.38	0.940
Inferior vena cava maximum diameter (cm)	0.92	± 0.19	0.81	± 0.20	0.93	± 0.24	0.089
Abdominal aorta diameter, diastole (cm)	0.80	± 0.09	0.77	± 0.07	0.84	± 0.07	0.020
Aortic proximal transverse arch diameter (cm)	1.54	± 0.19	1.47	± 0.17	1.55	± 0.18	0.385
Aortic distal transverse arch diameter (cm)	1.20	± 0.14	1.17	± 0.13	1.20	± 0.13	0.703
**Aortic isthmus diameter (cm)**	**1.12**	**1.05; 1.26**	**1.06***	**0.91; 1.10**	**1.14**	**0.99; 1.29**	**0.031**
**Aortic valve diameter (cm)**	**1.39**	**± 0.13**	**1.30*****	**± 0.07**	**1.40**	**± 0.11**	**<0.001**
Aortic sinus diameter (cm)	1.88	± 0.20	1.80	± 0.17	1.90	± 0.16	0.129
Aortic sinotubular junction diameter (cm)	1.59	1.50; 1.78	1.58	1.50; 1.73	1.64	1.51; 1.71	0.454
Pulmonary valve diameter (cm)	1.91	± 0.19	1.78	± 0.18	1.88	± 0.21	0.068
Left ventricle and atrium							
Interventricular septum dimension (cm)	0.4	0.4; 0.5	0.4	0.4; 0.5	0.5	0.4; 0.5	0.395
Left ventricular diastolic diameter (cm)	3.8	± 0.2	3.7	± 0.3	3.7	± 0.3	0.667
Left ventricular posterior wall dimension (cm)	0.5	0.4; 0.5	0.4	0.4; 0,5	0.5	0.4; 0.5	0.688
Left ventricular systolic diameter (cm)	2.6	2.4; 2.7	2.5	2.3; 2.8	2.5	2.4; 2.7	0.704
Left ventricular mass (g)	42	± 8	40	± 9	41	± 7	0.754
Left ventricular length (cm)	5.4	5.2; 5.8	5.4	5.1; 5.7	5.5	5.2; 6.0	0.420
**Left ventricular base (cm)**	**2.6**	**2.4; 2.7**	**2.5****	**2.3; 2.6**	**2.7**	**2.5; 2.9**	**0.001**
Left ventricular end-diastolic volume (ml)	41	± 8	38	± 6	44	± 8	0.076
Left ventricular end-systolic volume (ml)	17	± 3	16	± 3	19	± 4	0.125
**Left atrial volume (ml)**	**19**	**15; 21**	**17**	**14; 21**	**23***	**19; 26**	**0.006**
Right ventricle and atrium							
Right ventricular anterior wall thickness (cm)	0.3	0.3; 0.3	0.3	0.3; 0.4	0.3	0.3; 0.4	0.439
Right ventricular length (cm)	4.9	± 0.4	4.8	± 0.4	5.1	± 0.4	0.125
**Right ventricular base (cm)**	**2.6**	**± 0.2**	**2.5***	**± 0.2**	**2.7**	**±0.2**	**0.005**
Right ventricular mid-cavity (cm)	2.4	2.2; 2.5	2.3	2.2; 2.5	2.3	2.2; 2.7	0.354
**Right ventricular diastolic area (cm^2)**	**10.4**	**± 1.5**	**9.5***	**± 1.1**	**10.6**	**± 1.9**	**0.010**
Right ventricular systolic area (cm^2)	5.8	± 1.1	5.5	± 0.6	6.1	± 1.2	0.134
**Right atrial area (cm^2)**	**7.5**	**± 1.2**	**6.7****	**± 0.7**	**8.0**	**± 1.2**	**<0.001**

Data are given as mean ± SD or median Q1; Q3. P correspond to ANOVA or Kruskal-Wallis test, as appropriate. Significant differences in post-hoc tests (Dunnet, Games-Howell or Mann-Whitney U) between SGA or LGA and AGA are bolded and indicated with *, **, *** corresponding to two-sided significance levels of < 0.05, < 0.01, and <0.001, respectively. The significance level of Mann-Whitney U test is Bonferroni corrected for two group comparisons. *AGA* appropriate for gestational age; *LGA* large for gestational age; *SGA* small for gestational age.

Supplementary Table 3. Cardiac function for the AGA, SGA and LGA groups assessed from inferior vena cava and Doppler measurements

	AGA	(N = 48)	SGA	(N = 23)	LGA	(N = 19)	p
Inferior vena cava							
Respiratory change of inferior vena cava lumen diameter (%)	68	± 9	63	± 9	68	± 10	0.104
Pulmonary vein							
Pulmonary vein S-wave peak velocity (cm/s)	43	± 8	45	± 9	44	± 9	0.643
Pulmonary vein D-wave peak velocity (cm/s)	53	± 7	58	± 8	54	± 9	0.061
Pulmonary S/D -ratio (no unit)	0.81	0.73; 0.91	0.76	0.69; 0.88	0.80	0.71; 0.90	0.579
Mitral inflow Doppler							
**Mitral E-wave peak velocity (cm/s)**	**87**	**80; 99**	**99***	**90; 110**	**87**	**81; 98**	**0.013**
**Mitral A-wave peak velocity (cm/s)**	**40**	**32; 50**	**49****	**42; 55**	**41**	**34; 53**	**0.014**
Mitral E/A -ratio (no unit)	2.3	1.8; 2.7	1.9	1.8; 2.2	2.1	1.8; 2.7	0.461
Mitral inflow propagation velocity (cm/s)	63	± 14	59	± 12	59	± 13	0.380
Left ventricle pulsed wave tissue Doppler							
Lateral E'-wave peak velocity (cm/s)	18	± 3	18	± 3	19	± 2	0.343
Lateral E/E' -ratio (no unit)	5.1	± 1.0	5.6	± 1.2	4.7	± 0.6	0.020
Lateral A'-wave peak velocity (cm/s)	6	± 1	6	± 1	7	± 1	0.377
Lateral S'-wave peak velocity (cm/s)	9	8; 110	9	8; 10	10	9; 11	0.139
Septal E'-wave peak velocity (cm/s)	13	± 1	13	± 1	13	± 1	0.683
**Septal E/E' -ratio (no unit)**	**6.6**	**6.0; 8.1**	**7.5***	**7.0; 8.3**	**6.7**	**6.1; 7.0**	**0.007**
Septal A'-wave peak velocity (cm/s)	5	5; 6	5	5; 6	6	5; 6	0.019
Septal S'-wave peak velocity (cm/s)	8	± 1	7	± 1	8	±1	0.003
Tricuspid inflow Doppler							
Tricuspid E-wave peak velocity (cm/s)	50	± 8	54	± 7	50	± 8	0.128
Tricuspid A-wave peak velocity (cm/s)	36	± 8	36	± 6	39	± 10	0.615
Tricuspid E/A -ratio (no unit)	1.4	± 0.3	1.6	± 0.4	1.3	± 0.3	0.131
Right ventricle pulsed wave tissue Doppler							
Lateral E'-wave peak velocity (cm/s)	15	± 2	15	± 2	14	± 2	0.390
Tricuspid E/E' -ratio (no unit)	3.5	± 0.7	3.6	± 0.6	3.7	± 0.7	0.740
Lateral A'-wave peak velocity (cm/s)	9	± 2	8	± 2	9	±2	0.396
Lateral S'-wave peak velocity (cm/s)	13	± 2	13	± 2	13	± 1	0.600

Data are given as mean ± SD or median Q1; Q3. P correspond to ANOVA or Kruskal-Wallis test, as appropriate. Significant differences in post-hoc tests (Dunnet, Games-Howell or Mann-Whitney U) between SGA or LGA and AGA are bolded and indicated with *, ** corresponding to two-sided significance levels of < 0.05, and <0.001, respectively. The significance level of Mann-Whitney U test is Bonferroni corrected for two group comparisons. *AGA* appropriate for gestational age; *LGA* large for gestational age; *SGA* small for gestational age.

Supplementary Table 4. Speckle-tracking strain measurements for the AGA, SGA, and LGA groups.

	AGA	(N = 48)	SGA	(N = 23)	LGA	(N = 19)	p
Left ventricle circumferential strain[a]							
Left ventricular global basal circumferential peak strain (%)	-18.9	± 2.8	-20.0	± 3.2	-19.3	± 1.8	0.370
Left ventricular global basal circumferential systolic strain rate (1/s)	1.3	± 0.2	-1.4	± 0.2	-1.3	± 0.1	0.153
Left ventricular global basal circumferential early diastolic strain rate (1/s)	1.9	± 0.4	2.0	± 0.4	1.9	± 0.4	0.530
Left ventricular global basal circumferential late diastolic strain rate (1/s)	0.4	0.3; 0.5	0.5	0.3; 0.6	0.4	0.3; 0.5	0.420
Left ventricle longitudinal strainb							
Left ventricular global longitudinal peak strain (%)	-21.9	± 2.0	-21.2	± 1.7	-21.6	± 1.6	0.347
Left ventricular global longitudinal systolic strain rate (1/s)	-1.3	± 0.1	-1.2	± 0.2	-1.2	0.1	0.141
Left ventricular global longitudinal early diastolic strain rate (1/s)	2.6	± 0.4	2.7	± 0.3	2.5	± 0.3	0.532
Left ventricular global longitudinal late diastolic strain rate (1/s)	0.6	0.5; 0.7	0.6	0.6; 0.7	0.7	0.5; 0.7	0.406
Right ventricle free wall longitudinal strain[c]							
Right ventricular global longitudinal peak strain (%)	-29.8	± 4.5	-28.5	± 5.7	-29.8	± 5.3	0.729
Right ventricular global longitudinal systolic strain rate (1/s)	-1.9	± 0.4	-2.1	± 0.4	-1.9	± 0.5	0.475
Right ventricular global longitudinal early diastolic strain rate (1/s)	3.0	± 0.8	2.9	± 0.9	2.5	± 0.5	0.101
Right ventricular global longitudinal late diastolic strain rate (1/s)	1.0	± 0.4	0.9	± 0.5	1.2	± 0.7	0.479

Data are given as mean ± SD or median Q1; Q3. P correspond to ANOVA or Kruskal-Wallis test, as appropriate. *AGA* appropriate for gestational age; *LGA* large for gestational age; *SGA* small for gestational age.

[a]Measured from the parasternal short axis view at papillary muscle level from the parasternal short-axis views.

[b]Mean of measurements from the apical 4- and 2-chamber views.

[c]Measured from the free ventricular wall in the tilted apical 4-chamber view.

Supplementary Table 5. Univariate analyses to assess the effect sex, birth weight, anthropometrics, and adiposity on cardiac morphology

	α for BSA$^{\alpha}$	Male sex	Birth weight Z-score	Height (cm)	Height$^{2.16}$ ($m^{2.16}$)	Weight	Thoracic circumference (cm)	Lean body mass (kg)	BSA (m^2)	BSA$^{\alpha}$ (m^{α})	BMI (kg/m^2)	Body fat percentage (%)
		T-test, mean difference (SE)	r	r	r	r	r	r	r	r	r	r
Aorta and pulmonary artery												
Abdominal aorta diameter (cm)	0.50	0.034 (0.018)	**0.294****	0.209	na	**0.367*****	**0.339****	**0.326****	**0.352*****	**0.348****	**0.360*****	**0.222***
Aortic proximal transverse arch diameter (cm)	0.50	-0.036 (0.042)	0.131	0.103	na	**0.237***	0.209	**0.253***	**0.230***	**0.236***	**0.279***	0.164
Aortic distal transverse arch diameter (cm)	0.50	0.037 (0.030)	0.152	**0.307****	na	**0.282***	**0.219***	**0.347****	**0.299****	**0.301****	0.189	0.064
Aortic isthmus diameter (cm)	0.50	0.037 (0.038)	**0.312****	**0.360*****	na	**0.275***	**0.236***	**0.370*****	**0.305****	**0.308****	0.150	0.056
Aortic valve diameter (cm)	0.50	**0.067 (0.024)****	**0.293****	**0.412*****	na	**0.410*****	**0.371*****	**0.511*****	**0.435*****	**0.439*****	**0.295****	0.206
Aortic sinus diameter (cm)	0.50	**0.093 (0.040)***	0.184	**0.301****	na	**0.307****	**0.312****	**0.440*****	**0.326****	**0.331****	**0.227***	0.080
Aortic sinotubular junction diameter (cm)	0.50	0.037 (0.038)	0.120	**0.241***	na	**0.243***	**0.247***	**0.375*****	**0.257***	**0.260***	0.185	0.060
Pulmonary valve diameter (cm)	0.50	0.090 (0.046)	0.162	0.226	na	0.170	0.188	**0.265***	0.192	0.196	0.095	0.022
Left ventricle and atrium												
Left ventricular diastolic diameter (cm)	0.45	**0.214 (0.053)*****	-0.020	**0.412*****	na	**0.329****	**0.383*****	**0.427*****	**0.357*****	**0.359*****	0.188	0.030
Left ventricular mass (g)	1.25	**4.112 (1.674)***	0.068	**0.355*****	**0.351*****	**0.277****	**0.312****	**0.372*****	**0.307****	**0.303****	0.165	0.004
Left ventricle length (cm)	0.45	0.165 (0.093)	**0.225***	**0.337****	na	**0.370*****	**0.403*****	**0.437*****	**0.387*****	**0.389*****	**0.294****	0.135
Left ventricle base (cm)	0.45	0.049 (0.042)	**0.304****	**0.565*****	na	**0.550*****	**0.504*****	**0.599*****	**0.578*****	**0.581*****	**0.393*****	**0.320***
Left ventricle end-diastolic volume (ml)	1.30	**4.080 (1.580)***	**0.239***	**0.439*****	na	**0.427*****	**0.425*****	**0.534*****	**0.450*****	**0.448*****	**0.304****	0.117
Left atrium systolic volume (ml)	1.48	1.328 (0.920)	**0.332****	**0.421*****	na	**0.468*****	**0.442*****	**0.572*****	**0.487****	**0.480*****	**0.382*****	0.144
Right ventricle and atrium												
Right ventricle length (cm)	0.50	0.179 (0.091)	0.182	**0.344****	na	**0.303****	**0.341****	**0.415*****	**0.330****	**0.336****	0.204	0.089
Right ventricle base (cm)	0.50	0.064 (0.054)	**0.329****	**0.403*****	na	**0.275***	**0.293****	**0.404*****	**0.312****	**0.318****	0.130	0.106
Right ventricle mid-cavity (cm)	0.50	**0.169 (0.053)****	0.114	**0.445*****	na	**0.326****	**0.344****	**0.418*****	**0.356*****	**0.354*****	0.155	0.128
Right ventricle diastolic area (cm^2)	na	**1.039 (0.317)****	0.206	**0.511*****	na	**0.400*****	**0.421*****	**0.527*****	**0.440*****	na	**0.224***	0.130
Right atrium systolic area (cm^2)	na	0.501 (0.252)	**0.399*****	**0.499*****	na	**0.456*****	**0.468*****	**0.545*****	**0.489*****	na	**0.307****	0.198

Mean difference is reported as male – female (SE), reported r-values are Pearson's correlation coefficient. Significant associations are bolded, with *, **, *** corresponding to two-sided significance levels of < 0.05, < 0.01, and <0.001. The effect of age of was similarly explored using in univariate correlation analyses, with no significant correlations (not shown). The effect of systolic blood pressure and maternal pre-gestational diabetes on left ventricle mass was assessed with correlation analyses and Student's T-test, with neither showing a significant association (not shown). No significant associations were found for the non-normally distributed septal and left ventricular posterior wall dimensions and the right ventricular anterior wall dimensions, when examining the effect of sex using the Mann-Whitney U-test and the effect of birth weight, anthropometrics and adiposity using Spearman's correlation analysis (not shown). *BMI* body mass index and *BSA* body surface area.

Supplementary Table 6. Significant linear models assessing the effect of birth weight on cardiovascular dimensions.

Aorta and pulmonary artery	R^2	Model *P*	Predictor	Unstandardized β	Standardized B	p
Abdominal aorta diameter (cm)	0.158	0.003	Male sex	0.025	0.154	0.159
			Birth weight Z-score	0.007	0.219	0.070
			Lean body mass (kg)	0.007	0.186	0.135
Aortic distal arch transverse diameter (cm)	0.124	0.016	Male sex	0.017	0.064	0.568
			Birth weight Z-score	0.000	0.001	0.994
			**Lean body mass (kg)**	**0.021**	**0.332**	**0.010**
Aortic isthmus diameter (cm)	0.173	0.002	Male sex	0.010	0.029	0.789
			Birth weight Z-score	0.012	0.176	0.145
			**Lean body mass (kg)**	**0.024**	**0.298**	**0.017**
Aortic valve diameter (cm)	0.307	<0.001	Male sex	0.034	0.146	0.133
			Birth weight Z-score	0.005	0.104	0.324
			**Lean body mass (kg)**	**0.024**	**0.445**	**<0.001**
Aortic sinus diameter (cm)	0.216	<0.001	Male sex	0.046	0.123	0.236
			Birth weight Z-score	-0.001	-0.014	0.906
			**Lean body mass (kg)**	**0.038**	**0.427**	**<0.001**
Aortic sinotubular junction diameter (cm)	0.140	0.015	Male sex	0.001	0.003	0.977
			Birth weight Z-score	-0.004	-0.063	0.628
			**Lean body mass (kg)**	**0.032**	**0.400**	**0.004**
Left ventricle and atrium						
Left ventricle diastolic diameter (cm)	0.325	<0.001	**Male sex**	**0.156**	**0.292**	**0.003**
			**Birth weight Z-score**	**-0.026**	**-0.238**	**0.027**
			**Lean body mass (kg)**	**0.058**	**0.466**	**<0.001**
Left ventricle mass (g)	0.181	0.001	Male sex	2.565	0.160	0.136
			Birth weight Z-score	-0.391	-0.118	0.316
			**Lean body mass (kg)**	**1.459**	**0.391**	**0.002**
Left ventricle length (cm)	0.200	<0.001	Male sex	0.098	0.111	0.287
			Birth weight Z-score	0.007	0.037	0.749
			**Lean body mass (kg)**	**0.080**	**0.389**	**0.001**
Left ventricle base (cm)	0.363	<0.001	Male sex	-0.013	-0.033	0.723
			Birth weight Z-score	0.003	0.032	0.752
			**Lean body mass (kg)**	**0.055**	**0.594**	**<0.001**
Left ventricle end-diastolic volume (ml)	0.305	<0.001	Male sex	2.330	0.152	0.119
			Birth weight Z-score	0.038	0.012	0.911
			**Lean body mass (kg)**	**1.741**	**0.489**	**<0.001**
Left atrium systolic volume (ml)	0.331	<0.001	Male sex	0.510	0.060	0.531
			Birth weight Z-score	0.115	0.066	0.532
			**Lean body mass (kg)**	**1.039**	**0.524**	**<0.001**
Right ventricle and atrium						
Right ventricle length (cm)	0.187	<0.001	Male sex	0.103	0.119	0.259
			Birth weight Z-score	-0.001	-0.007	0.951
			**Lean body mass (kg)**	**0.079**	**0.390**	**0.002**
Right ventricle base (cm)	0.192	<0.001	Male sex	0.027	0.054	0.605
			Birth weight Z-score	0.019	0.182	0.115
			**Lean body mass (kg)**	**0.036**	**0.307**	**0.011**
Right ventricle mid-cavity (cm)	0.227	<0.001	**Male sex**	**0.118**	**0.225**	**0.031**
			Birth weight Z-score	-0.006	-0.056	0.617
			**Lean body mass (kg)**	**0.047**	**0.386**	**0.001**
Right ventricle diastolic area (cm^2)	0.327	<0.001	**Male sex**	**0.649**	**0.207**	**0.033**
			Birth weight Z-score	-0.017	-0.026	0.807
			**Lean body mass (kg)**	**0.360**	**0.494**	**<0.001**
Right atrium systolic area (cm^2)	0.336	<0.001	Male sex	0.266	0.111	0.245
			Birth weight Z-score	0.096	0.193	0.066
			**Lean body mass (kg)**	**0.240**	**0.429**	**<0.001**

Significant associations are bolded.
